# Fusarium oxysporum Disrupts Microbiome-Metabolome Networks in Arabidopsis thaliana Roots

**DOI:** 10.1128/spectrum.01226-22

**Published:** 2022-06-29

**Authors:** Enoch Narh Kudjordjie, Kourosh Hooshmand, Rumakanta Sapkota, Behrooz Darbani, Inge S. Fomsgaard, Mogens Nicolaisen

**Affiliations:** a Department of Agroecology, Faculty of Technical Sciences, Aarhus University, Slagelse, Denmark; State Key Laboratory of Mycology, Institute of Microbiology, Chinese Academy of Sciences

**Keywords:** plant pathogen, root microbiome, glucosinolates, phenylpropanoids, phytohormones, microbial co-occurrence networks, plant resistance

## Abstract

While the plant host metabolome drives distinct enrichment of detrimental and beneficial members of the microbiome, the mechanistic interomics relationships remain poorly understood. Here, we studied microbiome and metabolome profiles of two Arabidopsis thaliana accessions after Fusarium oxysporum f.sp. *mathioli* (FOM) inoculation, *Landsberg erecta* (Ler-0) being susceptible and Col-0 being resistant against FOM. By using bacterial and fungal amplicon sequencing and targeted metabolite analysis, we observed highly dynamic microbiome and metabolome profiles across FOM host progression, while being markedly different between FOM-inoculated and noninoculated Col-0 and Ler-0. Co-occurrence network analysis revealed more robust microbial networks in the resistant Col-0 compared to Ler-0 during FOM infection. Correlation analysis revealed distinct metabolite-OTU correlations in Ler-0 compared with Col-0 which could possibly be explained by missense variants of the *Rfo*3 and *Rlp*2 genes in Ler-0. Remarkably, we observed positive correlations in Ler-0 between most of the analyzed metabolites and the bacterial phyla Proteobacteria, Bacteroidetes, Planctomycetes, Acidobacteria, and Verrucomicrobia, and negative correlations with Actinobacteria, Firmicutes, and Chloroflexi. The glucosinolates 4-methyoxyglucobrassicin, glucoerucin and indole-3 carbinol, but also phenolic compounds were strongly correlating with the relative abundances of indicator and hub OTUs and thus could be active in structuring the *A. thaliana* root-associated microbiome. Our results highlight interactive effects of host plant defense and root-associated microbiota on Fusarium infection and progression. Our findings provide significant insights into plant interomic dynamics during pathogen invasion and could possibly facilitate future exploitation of microbiomes for plant disease control.

**IMPORTANCE** Plant health and fitness are determined by plant-microbe interactions which are guided by host-synthesized metabolites. To understand the orchestration of this interaction, we analyzed the distinct interomic dynamics in resistant and susceptible *Arabidopsis* ecotypes across different time points after infection with Fusarium oxysporum (FOM). Our results revealed distinct microbial profiles and network resilience during FOM infection in the resistant Col-0 compared with the susceptible Ler-0 and further pinpointed specific microbe-metabolite associations in the Arabidopsis microbiome. These findings provide significant insights into plant interomics dynamics that are likely affecting fungal pathogen invasion and could possibly facilitate future exploitation of microbiomes for plant disease control.

## INTRODUCTION

Plant-microbe interactions are of high agronomic importance for their beneficial or detrimental effects on plant growth and productivity. For a holistic understanding of these interactions, numerous recent studies have attempted to unravel the complex interactions occurring between plants and their associated microbiota. Together with environmental factors, host traits, including plant metabolites mediate the assembly of the plant microbiota (1, 2) by for instance serving as carbon and energy sources or as chemical signaling molecules (3). Strong evidence exists that plant metabolites shape the host-associated microbiome (4–6). For example, coumarins, flavonoids, glucosinolates, benzoxazinoids, and phytohormones such as salicylic acid (SA), jasmonic acid (JA), and ethylene play profound roles in modulating root-associated microbiomes (5, 7–12).

During pathogen invasion, host metabolic reconfiguration mostly follows immune signaling events triggered by the recognition of pathogenic signatures (13). A number of studies have reported changes in metabolic compounds after pathogen infection, followed by alterations in microbiomes composition (14–16). The composition and diversity of the host-associated microbiota determine pathogen invasion resistance (17–19). Highly complex and diverse microbial communities characterized by a web of cooperative and antagonistic interactions among microbial members (20, 21) have been shown to be more resistant to pathogenic perturbations (22, 23). The host-associated microbiota suppresses the invasion of pathogens by antagonizing effects or through the activation of host-defense mechanisms (24, 25). Ecological network metrics are used to predict the mechanisms of persistence and stability of microbial communities (17, 26). For example, Wei et al. (26), showed that bacterial community networks having low nestedness and high connectance reduced the invasion success and progression of Ralstonia solanacearum in tomato roots. Moreover, network analysis has highlighted ecologically important microorganisms such as indicator species or hub members in microbial communities (21, 27–29). Hub species have, by definition, many network connections, so their removal destabilizes the overall network structure (27) and thereby affect community resilience to pathogen perturbations.

Because resistance to pathogen infection is likely a combination of plant and microbiota traits, it is equally important to gain insights into microbial community responses to pathogen invasion for efficient disease control. Novel plant-mediated avenues for designing pathogen-resilient plant microbiomes require in-depth knowledge of the complex and dynamic interactions occurring between the metabolome and microbial communities. However, studies deciphering links between soilborne pathogens, microbiota, and plant-metabolites are limited.

The Arabidopsis thaliana (hereafter, *Arabidopsis*) ecotypes Col-0 and Ler-0 have differential resistance responses to species of the genus Fusarium (30–33). Fusarium oxysporum is a hemi-biotrophic root pathogen with a broad host range infecting several plants species, including the model plant *Arabidopsis* (30). *Arabidopsis* ecotypes exhibit differential resistance levels to Fusarium oxysporum *f.* sp. *matthiolae* (FOM), one of the *formae speciales* causing disease in the crucifers (30). This makes the *Arabidopsis*-FOM system an ideal model for studying plant-pathogen interactions. The resistance to Fusarium oxysporum in *Arabidopsis* Col-0 is controlled by six QTLs (26), located in genes encoding the RFO1 (26), RFO2 (31), RFO3 (34), and RLP2 (31) proteins.

We hypothesized that Fusarium oxysporum pathogenicity and its impact on the Arabidopsis microbiome and metabolome is dependent on the levels of disease resistance. We further assumed that a successful FOM infection would result in more significant global shifts in the microbiomes and metabolomes of the susceptible Ler-0 than in the resistant Col-0. The objectives of our study were to (i) understand how FOM infection influences root-associated microbial community structures and *Arabidopsis* metabolite profiles, (ii) examine the interomic dynamics during FOM infection in the two *Arabidopsis* accessions with different resistance profiles, and (iii) assess microbiome-metabolome associations occurring in the inoculated and noninoculated Col-0 and Ler-0 lines. To test our hypotheses, we studied omics profiles in a time series of FOM infection in Col-0 and Ler-0.

## RESULTS

We summarize the response of Arabidopsis to FOM and the omics dynamics during resistant and susceptible interactions. The quantified metabolites and their metabolic pathways are shown (Table S1; Fig. S1A, B). All supplemental materials are provided in this link https://doi.org/10.6084/m9.figshare.19422260.v1.

### FOM colonizes the susceptible Ler-0 faster than the resistant Col-0.

**FIG 1:**
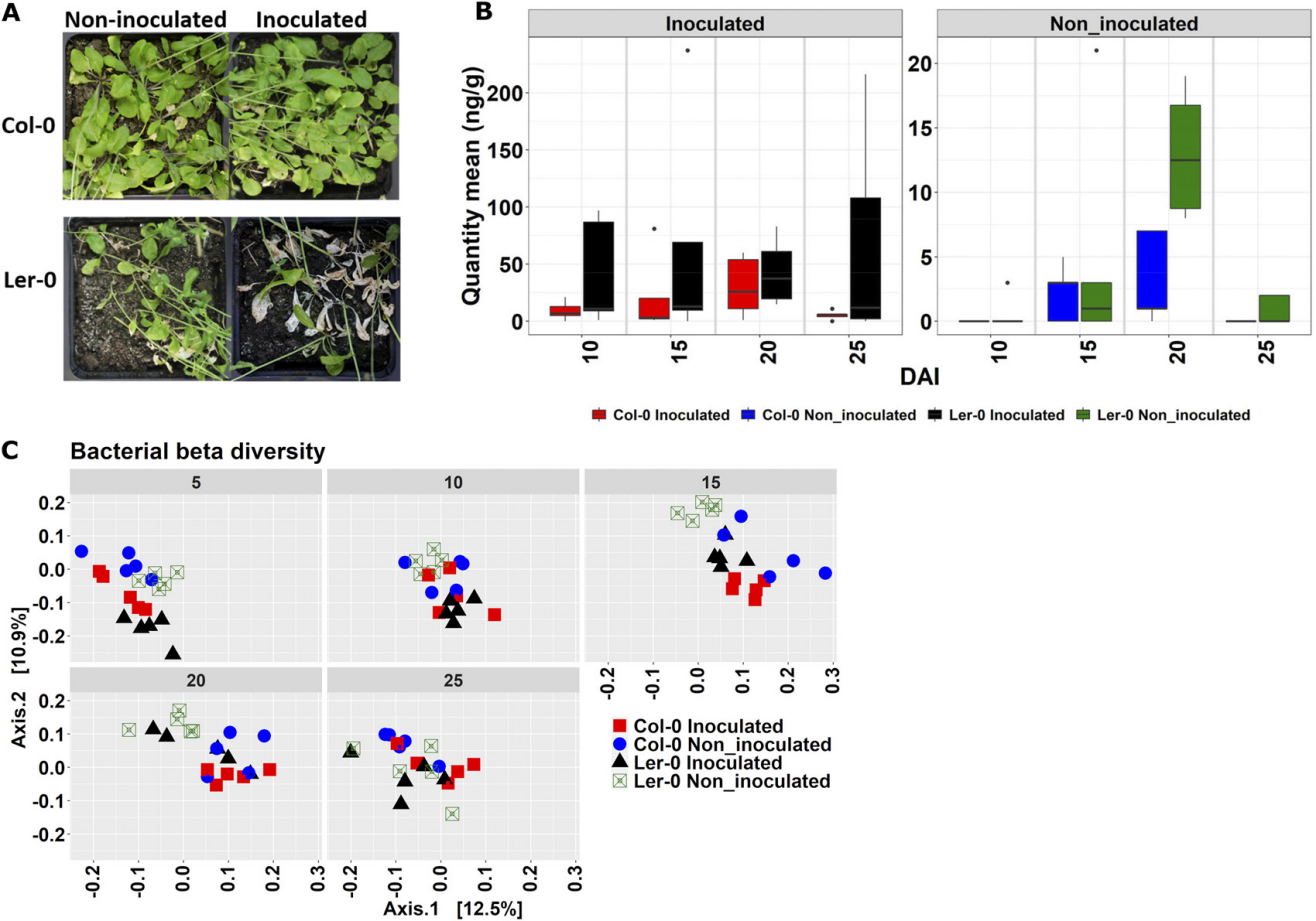
Fusarium oxysporum f.sp. *mathioli* (FOM) symptoms, quantity, and effects on *Arabidopsis* root-associated microbiomes. (A) The *Arabidopsis* genotypes Col-0 and Ler-0 showing wilting symptoms caused by FOM at 25 days after inoculation (DAI). (B) Boxplot of quantitative PCR (qPCR) data of the abundance of Fusarium oxysporum in stems of noninoculated and inoculated Col-0 and Ler-0 samples at 10, 15, 20, and 25 DAI. Figure A and data for Figure B have been published in Hooshmand et al. (35). (C) Principal-coordinate analysis (PCoA) of *Arabidopsis* root bacterial communities across DAI using Bray-Curtis dissimilarity distances.

After FOM inoculation, the most remarkable symptoms of wilting were observed in Ler-0, were strongest at 25 days after inoculation (DAI) (Fig. 1A). Symptoms of wilting characteristic of F. oxysporum infection were also observed in noninoculated Ler-0 (Fig. 1A). qPCR confirmed significantly higher F. oxysporum quantities in inoculated Ler-0 compared with the noninoculated Ler-0 samples (Fig. S2A). No significant differences were observed between inoculated and noninoculated samples at the individual DAIs, although F. oxysporum levels were generally higher in the inoculated samples (Fig. 1B). F. oxysporum DNA was detected at low levels in the noninoculated plants, most likely due to some presence of F. oxysporum in the soil used for the experiments. F. oxysporum DNA levels increased at a slower rate in Col-0 and declined at 25 DAI (Fig. 1B).

### Microbiome structures.

We characterized the Col-0 and Ler-0 root-associated (roots and finely attached soils) microbiomes during FOM progression. We obtained a total of 2,138,612 bacterial sequence reads (range 1,420 to 72,161; median 16,985 per sample) resulting in 8,359 operational taxonomic units (bOTUs) while the fungal community profiling yielded 2,995,948 reads (range 11,192 to 37,255; median 18,122 per sample) resulting in 438 fungal OTUs (fOTUs) (Table S2). The microbial read distribution and rarefaction curves are reaching an asymptote, indicating satisfactory representation of the most common microbes in the studied samples (see Fig. S2B to E). Relative abundances at class and genus levels for bacteria and fungi, respectively, are shown (Fig. S3A, B). The bacterial classes Alpha/Gamma-proteobacteria and Actinobacteria were the dominant taxa in both inoculated and noninoculated samples. Fungal reads from FOM-inoculated samples were, not surprisingly, dominated by Fusarium oxysporum (fOTU1), as also supported by qPCR (Fig. S3C, D), and the appearance of symptoms (Fig. 1A).

We assessed the impact of DAI, host genotype, and FOM treatment on the bacterial and fungal communities. Bacterial alpha diversity was significantly higher in noninoculated than inoculated Ler-0 samples at 5, 10, and 15 DAIs (Fig. S3E). Bacterial and fungal communities showed distinct clustering across the different DAIs in PCoA plots according to genotype and FOM inoculation (Fig. 1C [bacterial communities] and Fig. S3F [fungal communities]). Bacterial communities were clearly separated based on FOM treatment in Ler-0 at early DAIs but became indistinguishable at later stages. A permutational multivariate analysis of variance (PERMANOVA) showed that in the whole data set, DAI had the highest effect on bacterial communities (Adonis; R^2^ = 0.17, *P < *0.001, Table 1), while FOM inoculation, not surprisingly, explained the highest variation in fungal communities (Adonis; R^2^ = 0.37, *P < *0.001) (Table 1). By subsetting data sets, DAI was having the strongest effect on bacterial communities in the inoculated Col-0 (Adonis; R^2^ = 0.42, *P < *0.001) (Table S3), and on fungal communities in the inoculated Ler-0 (Adonis; R^2^ = 0.59, *P < *0.001) (Table S3). Using the data sets partitioned for individual DAIs, the strongest effect of FOM inoculation was at 5 and 10 DAI (Table 1). The effect of FOM inoculation diminished with time, whereas the genotype effect on fungal community increased with increasing DAI it had the highest effect on the bacterial communities at 20 DAI (Table 1).

**TABLE 1:** Summary of permutational analysis of variance (PERMANOVA) using the “adonis” test on Bray-Curtis distance matrices for bacterial and fungal community dissimilarity assessment using 1,000 permutations

Dataset	Factor	Bacteria R2	Fungi R2
Whole	Genotype	0.02***^a^	0.05***
	DAI	0.17***	0.08***
	FOM inoculation	0.06***	0.37***
	Genotype*DAI	0.10***	0.07***
	Genotype* FOM inoculation	ns^*b*^	0.02***
	DAI* FOM inoculation	0.05***	0.08***
	Genotype*DAI* FOM inoculation	0.04**	0.05***
DAI			
5	Genotype	0.13***	0.04*
	FOM inoculation	0.20***	0.69***
	Genotype * FOM inoculation	ns	ns
10	Genotype	0.13***	ns
	FOM inoculation	0.15***	0.60***
	Genotype * FOM inoculation	ns	ns
15	Genotype	0.15***	0.11**
	FOM inoculation	0.14***	0.41***
	Genotype * FOM inoculation	0.07*	0.10**
20	Genotype	0.17***	0.16***
	FOM inoculation	0.09*	0.46***
	Genotype * FOM inoculation	ns	0.10**
25	Genotype	0.14***	0.32***
	FOM inoculation	ns	0.21***
	Genotype * FOM inoculation	0.08*	0.12**

a Significance of test indicated as ***, *P* < 0.001; **, *P* > 0.01; *, *P* < 0.05.

b The ns denotes not statistically significant and R^2^ is the proportion of variation explained.

### FOM inoculation and host resistance affect OTUs.

For an overview of microbial relative read abundances in the treatments, a heatmap visualization of the 50 most abundant bacterial and fungal OTUs is shown (Fig. S4A, B). In the bacterial data set, we observed a remarkable enrichment of bOTU6 (*Rhizobium*) and bOTU10 (Pseudomonas protegens) in inoculated samples (Fig. S4C). Also, the relative abundances of bOTU4 (*Streptomyces*) was found to be significantly higher in Col-0 that in Ler-0 and depleted during FOM infection in both genotypes (Fig. S4D). Differential analysis revealed significantly different OTUs in Col-0 and Ler-0 samples. bOTU99 (*Flavobacterium*) and bOTU248 (*Pedobacter*) were the most enriched in Col-0 and Ler-0, respectively (Fig. S5A). Both fOTU14 and fOTU39 (both *Cladosporium*) and *Rhizophlyctis* (fOTU267) were the most enriched fungal genera in noninoculated Col-0 and Ler-0, respectively (Fig. S5B). In contrast, the family Leptosphaeriaceae (fOTU57) and an uncultured Agaricomycetes (fOTU43) were strongly enriched in inoculated Ler-0 and Col-0, respectively. bOTU6 (*Rhizobium*) and bOTU10 (Pseudomonas protegens) but also bOTU65 (*Delftia*), bOTU94 (*Stenotrophomonas*) and fOTU1 (FOM) were found to be strongly enriched in inoculated samples compared with noninoculated samples (Fig. S5C, D).

**FIG 2:**
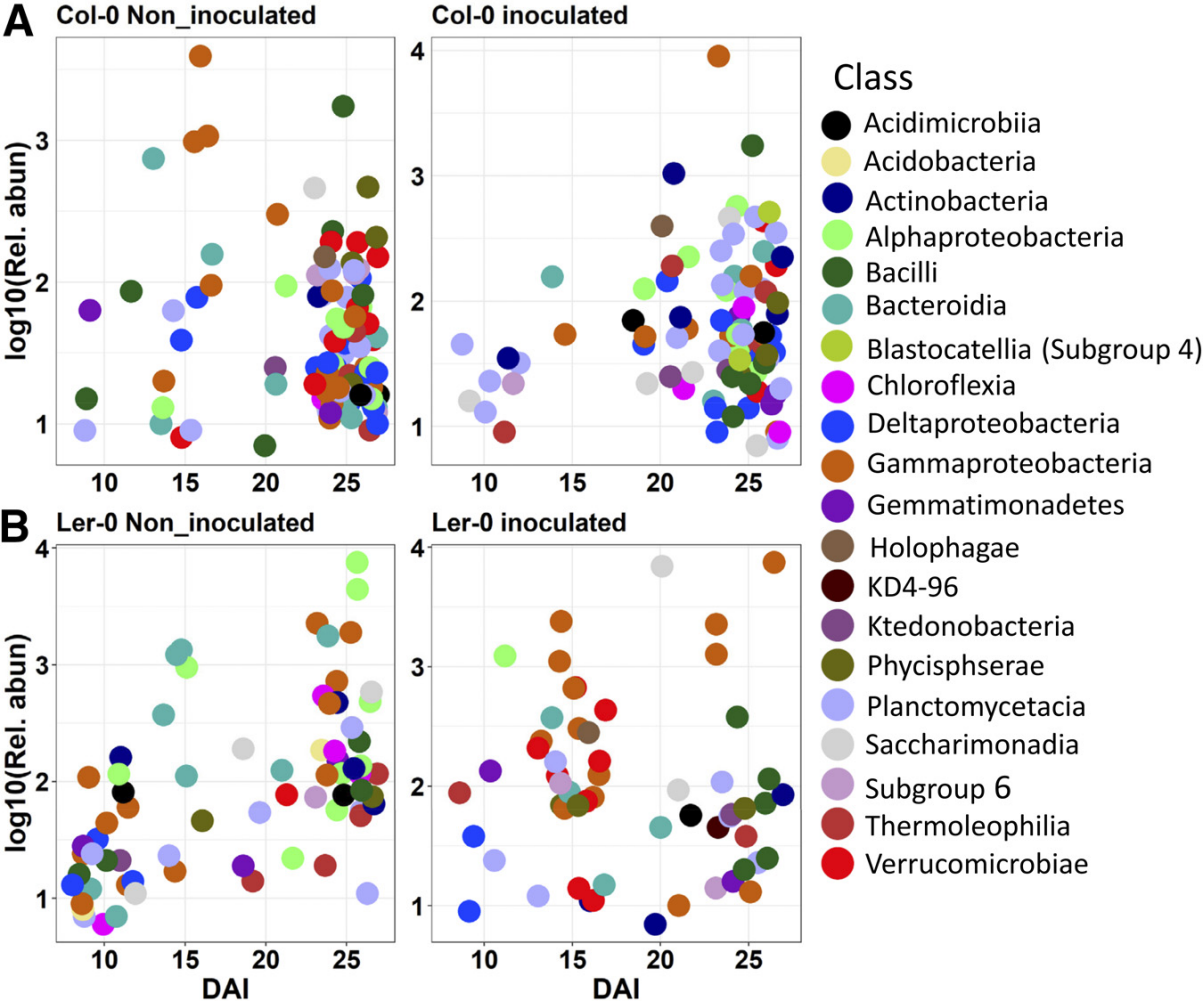
Enrichment of bacterial indicator OTUs across different DAIs. Bacterial indicator OTU enrichment in noninoculated and inoculated samples of (A) Col-0 and (B) Ler-0 at different days after inoculation (DAI). Highly significant OTUs (*P* < 0.01) with an indicator value of at least 0.4 was used to define indicator species.

Indicator species analysis identified the highest numbers of indicator bOTUs in Col-0, especially in the noninoculated samples (Fig. S6A; inserted table; Table S5). Most of the indicator bOTUs belonged to Proteobacteria and Actinobacteria, while Sordariomycetes, Eurotiomycetes, and Dothidiomycetes distinctively dominate inoculated and noninoculated samples (Fig. S6A, B). Actinobacteria and Planctomycetes were strongly enriched in inoculated Col-0. Similarly, while Alphaproteobacteria was depleted, Gammaproteobacteria and Verrucomicrobiae were highly enriched in inoculated versus noninoculated Ler-0. Furthermore, we found distinct patterns of enrichment of indicator OTUs across different DAIs, particularly within bOTUs (Fig. 2; Table S5). In Col-0, indicator bOTUs were most actively recruited at 25 DAI, while indicator bOTU numbers moderately increased in Ler-0 with increasing DAIs (Fig. 2A, B). bOTU10 (P. protegens) was the most abundant indicator in inoculated Col-0 and Ler-0, while bOTU61 (*Duganella*) was highly depleted in inoculated Col-0, and bOTU6 (*Rhizobium*) was depleted in inoculated Ler-0 (Table S5). There were weaker patterns of enrichment of fOTU indicators across DAI, peaking at 25 DAI in Col-0 (Fig. S6C; Table S5).

### Microbial networks break down in the susceptible Ler-0 after FOM inoculation.

**FIG 3:**
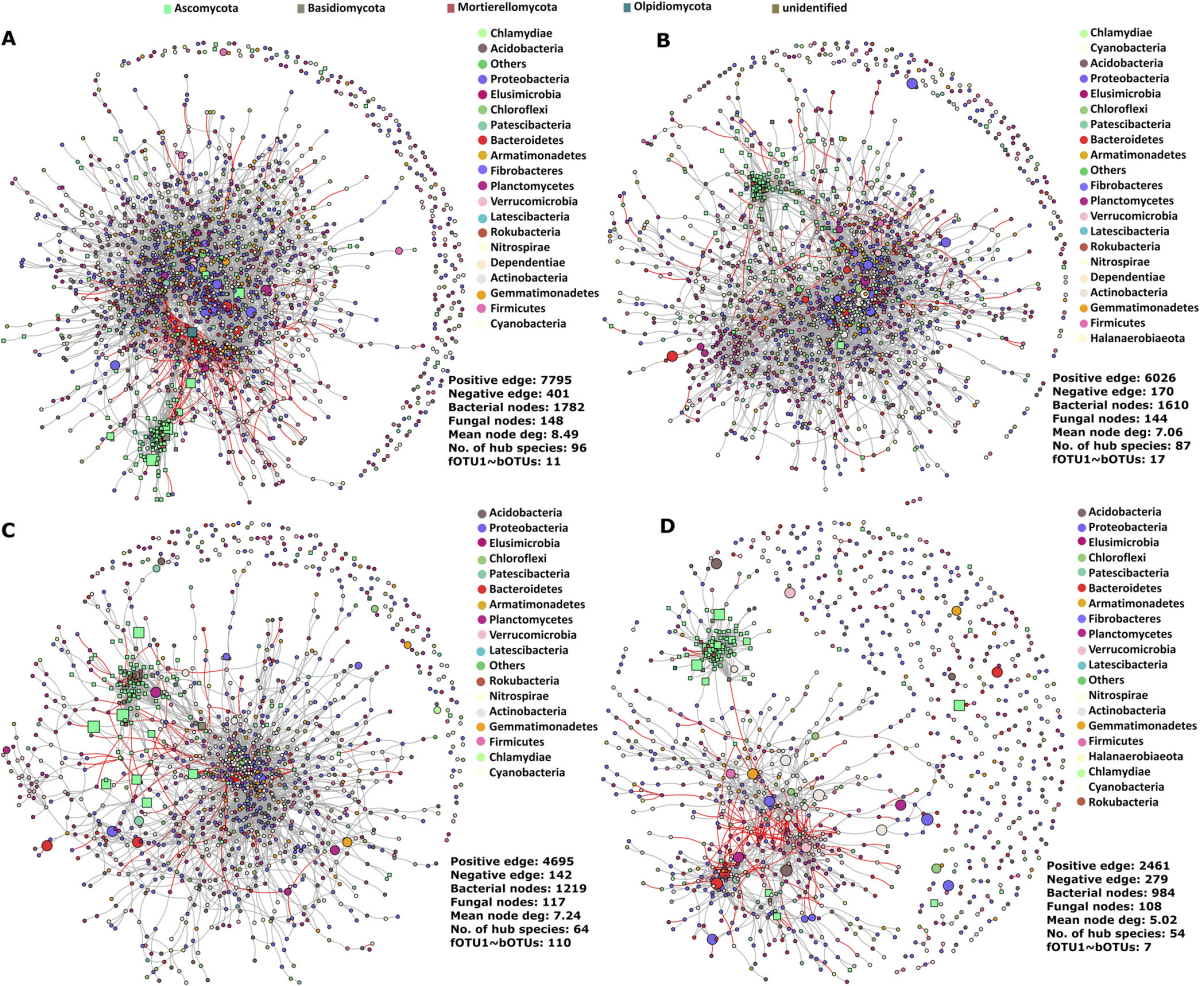
Microbial co-occurrence networks of Col-0 and Ler-0. Networks depicting bacterial and fungal inter- and intrakingdom interactions in noninoculated Col-0 (A) and Ler-0 (B) and the inoculated Col-0 (C) and Ler-0 (D). Positive and negative correlations are shown as gray and red edges, respectively. Bacterial and fungal nodes are represented as square and circle symbols in the network, respectively. Indicator OTUs are shown as larger nodes.

Co-occurrence networks visualized microbial co-occurrences and highlighted indicator OTUs in the overall OTU networks (Fig. 3; Table S6). Network robustness determined by node degree was highest in the noninoculated networks (Col-0: 8.49; Ler-0: 7.24) compared with the inoculated networks (Col-0: 7.06; Ler-0: 5.02). Communities in noninoculated samples had more nodes and edges, while inoculated Ler-0 had the highest relative number of negative edges (Fig. 3). Networks of Col-0 had a dense core cluster with most of the indicator bOTUs found within this cluster (Fig. 3A, B). In contrast, Ler-0 networks were less dense and had smaller microbial clusters especially in inoculated samples. Indicator fOTUs were mostly located outside the main clusters, particularly in inoculated samples (Fig. 3C, D).

The total number of positive co-occurrences were notably smaller in inoculated samples (Ler-0, 2740; Col-0, 6196) than in noninoculated samples (Ler-0, 4837; Col-0, 8196), and was smallest in Ler-0. (Table S6). A remarkably higher proportion of negative co-occurrences was observed in inoculated Ler-0 (11.3%) compared with the other treatments (2.8% to 5.1%) (Fig. 3).

The number of hub OTUs (representing the 5% OTUs having the highest numbers of connections to other OTUs) were highest in the networks of noninoculated samples (Fig. 3; Table S6). Microbial OTUs acting as both indicators and hub members were distinct and varied in numbers in inoculated and noninoculated genotypes. Some of the most highly connected hubOTUs included Caulobacteraceae), bOTU998 (Xanthomonadaceae), bOTU885 (Chitinophagaceae), bOTU188 (Mycobacteriaceae), bOTU4664 (Xanthobacteraceae), bOTU15 (Xanthobacteraceae), bOTU12 (Nocardioidaceae), bOTU 27 (Methyloligellaceae), fOTU6 (*Fusicolla*), fOTU8 (Chaetomiaceae), and fOTU29 (Piskurozymaceae) (Table S6).

Finally, the highest number of bOTUs that were negatively correlating with FOM (fOTU1) (Spearman’s *r *> 0.05, *P* < 0.05) was observed in noninoculated Ler-0 samples mostly, including taxa belonging to Proteobacteria, Actinobacteria, and Planctomycetes (Table S7).

### FOM infection alters metabolite profiles in *Arabidopsis*.

**FIG 4:**
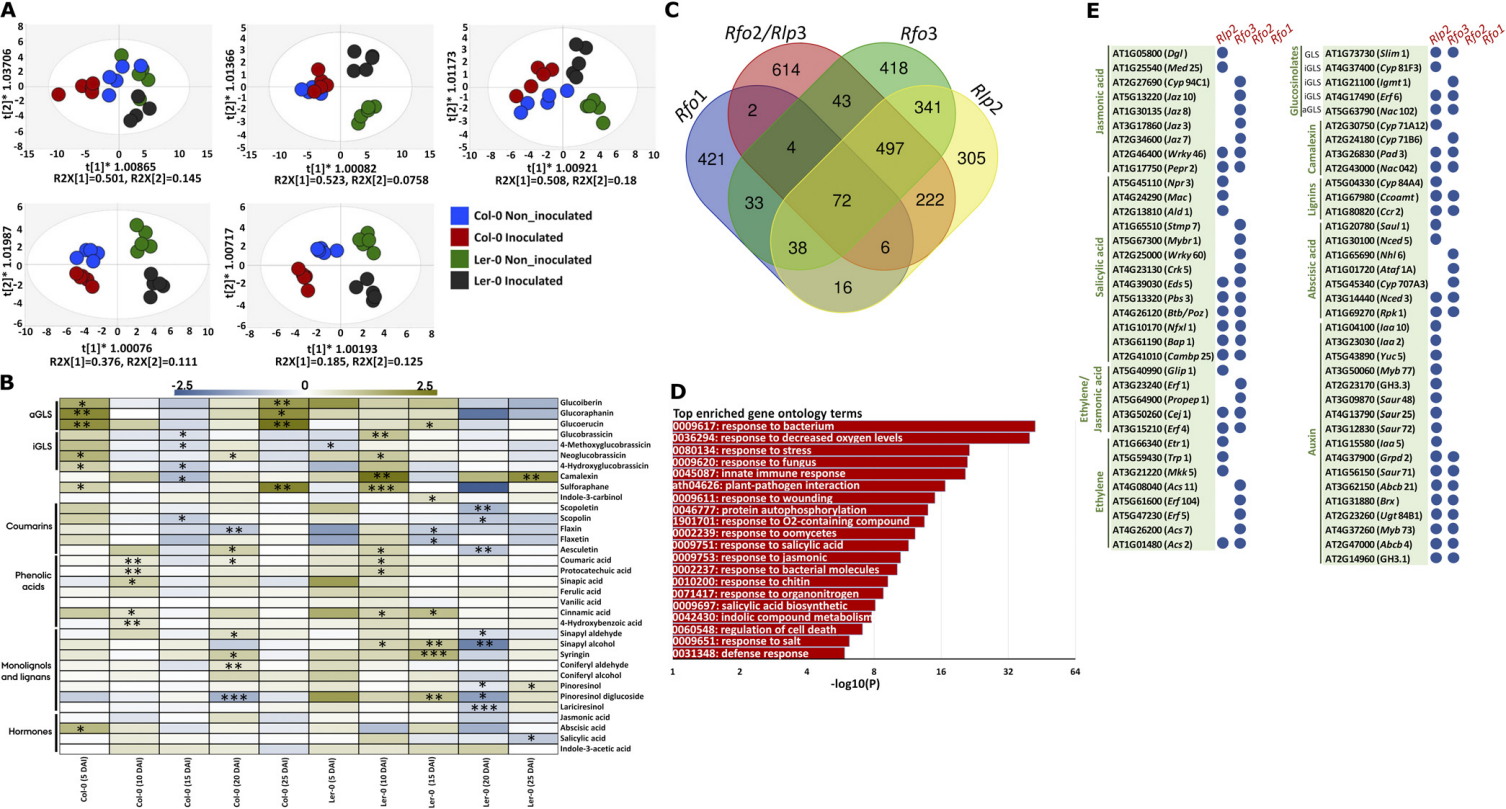
Metabolite data-derived OPLS-DA, heatmap representation of the metabolites identified in the roots of *Arabidopsis* Col-0 and Ler-0 after Fusarium oxysporum f.sp. *mathioli* (FOM) inoculation at different days after inoculation (DAI). (A) OPLS-DA score plots at different DAIs for inoculated and noninoculated Col-0 and Ler-0. Hotelling’s T2 statistical test cut-off was set at 95% to identify extreme outliers. (B) A heatmap comparison of secondary metabolites; glucosinolates (aliphatic glucosinolates [aGLS], indolic glucosinolates [iGLS], glucosinolates hydrolysis products, coumarins, phenolic acids, monolignols, lignans, and hormones) with log_2-fold_ changes (mean inoculated/mean noninoculated) and significance levels in inoculated and noninoculated Col-0 and Ler-0 (*n* = 5) at different DAI. Yellow color intensity representing higher values compared with noninoculated, and blue lower values compared with noninoculated. Significant differences between inoculated and noninoculated samples are indicated by asterisks (*, *P < *0.05; **, *P < *0.01; ***, *P < *0.001). (C) Fusarium resistance genes and the numbers of their coexpressors. Publicly available transcriptome-data comprising >1,000 experiments on Arabidopsis thaliana were used and co-regulated genes were selected with a Pearson’s correlation coefficient higher than 0.75 and a Mutual Rank of at least 2.2. (D) Pathway enrichment analysis highlights different pathogen response mechanisms in which the co-expressed genes are involved. (E) The *Rlp*2 and *Rfo*3 specific co-regulated genes which are involved in glucosinolate, camalexin, lignin, jasmonic acid, salicylic acid, ethylene, abscisic acid, and auxin biosynthesis and signaling. *Rlp*2, AT1G17240; *Rfo*3, AT3G16030; *Rfo*2, AT1G17250; *Rfo*1, AT1G79670.

We profiled a range of root metabolites that are supposedly playing roles in plant-microbe interactions in *Arabidopsis* (Table S8). Orthogonal partial least-squares discriminant analysis (OPLS-DA) showed increasingly distinct clustering of the treatment groups across time (Fig. 4). FOM inoculation affected Ler-0 metabolites from the onset of the experiment, while inoculation did not notably affect Col-0 metabolites at the early time points but formed separate clusters at later stages (Fig. 4A). There were significant temporal and cultivar-specific changes in levels of the individual metabolites. Ler-0 and Col-0 varied in their glucosinolate (GLS) content after FOM inoculation. Almost 98% of the total GLS detected in Ler-0 were indolic GLSs (iGLS), whereas Col-0 contained higher concentrations of aliphatic GLSs (aGLS) (10% to 30% of the total GLS). FOM inoculation of Col-0, but not Ler-0, increased levels of glucoraphanin, sulforaphane, glucoerucin, and glucoiberin in roots 5 DAI (approximately 4-fold increases) as well as 25 DAI (4-, 10-, and 3.5-fold increases, respectively) (Fig. 4B; Table S8).

In inoculated Col-0 roots, neoglucobrassicin and 4-hydroxyglucobrassicin concentrations increased 2- to 3-folds at 5 DAI, while levels of glucobrassicin, 4-methoxyglucobrassicin and 4-hydroxyglucobrassicin decreased at 15 DAI (2-fold). A significant increment in the levels of several indole glucosinolates occurred in Ler-0 inoculated roots at 10 DAI. An increase in camalexin concentrations in response to FOM inoculation occurred at a much greater extent in Ler-0 compared with Col-0, especially at 10 (8-fold), 20 (2.5-fold), and 25 DAI (4-fold). Among the quantified lignans and lignan precursors, sinapaldehyde, sinapyl alcohol, and pinoresinol diglucoside increased in inoculated Ler-0 at 15 DAI, while sinapaldehyde, sinapyl alcohol, pinoresinol diglucoside, lariciresinol, and pinoresinol decreased and coumaric acid, sinapaldehyde, coniferyl aldehyde, and syringin increased in inoculated Col-0 at 20 DAI. Among the hormones investigated, a significant increase (2.5-fold) in the concentration of abscisic acid in Col-0 occurred at 5 DAI. In addition, the level of SA decreased significantly in roots of inoculated Ler-0 at 25 DAI.

### *Arabidopsis* resistance genes and the metabolome.

To infer the relationship between FOM resistance genes and metabolites, we analyzed genes involved in Fusarium resistance using genomic and transcriptomic data. We identified ~3,000 genes co-expressing with at least one of the known Fusarium resistance genes *Rfo*1, *Rfo*2, *Rfo*3, and *Rlp*2 (30, 35) (Fig. 4C). Our analysis mapped these co-expressed genes to pathogen response pathways, e.g., responses to SA and JA, GLS metabolic processes, and xenobiotic detoxification (Fig. 4D; Table S9). We found 72 genes that were co-expressed with all four resistance genes, of which the indole-glucosinolate biosynthesis related genes *Igmt*3 (AT1G21110), *Pen*2 (AT2G44490), *Pen*3 (AT1G59870), *Cad*1/*Pcs*1 (AT5G44070), the salicylic acid signaling related genes *Npr*1 (AT1G64280), *Wakl*10 (AT1G79680), and the pathogen-associated molecular pattern triggered genes *Exo*70B2 (AT1G07000), Pub23 (AT2G35930), and Pub24 (PUB24) were notable. Next, we analyzed the resistance genes and found many missense variants in RFO3 and RLP2 of Ler-0 while RFO1 and RFO2 were identical between the ecotypes (Fig. S7). Based on these observations, we analyzed the *Rfo*3 and *Rlp*2 specific responses against Fusarium that are conceivably perturbed in Ler-0 due to the missense mutations. Interestingly, we identified *Rfo*3 and *Rlp*2-specific co-regulated genes involved in jasmonic acid, salicylic acid, abscisic acid, ethylene, and auxin biosynthesis and signaling as well as biosynthesis of lignins, indol-glucosinolates, and aliphatic-glucosinolate (Fig. 4E).

### Distinct metabolite-OTU correlations in Col-0 and Ler-0.

**FIG 5:**
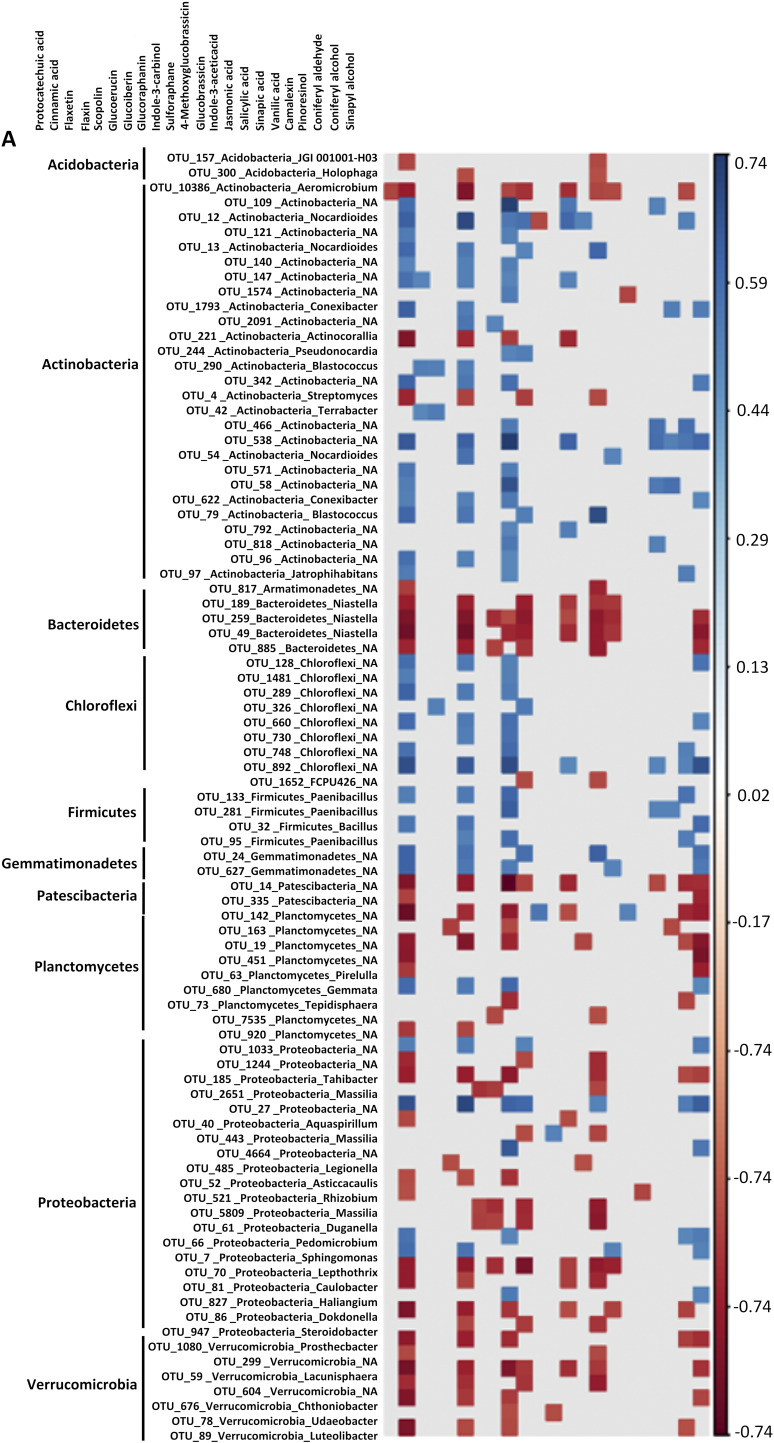

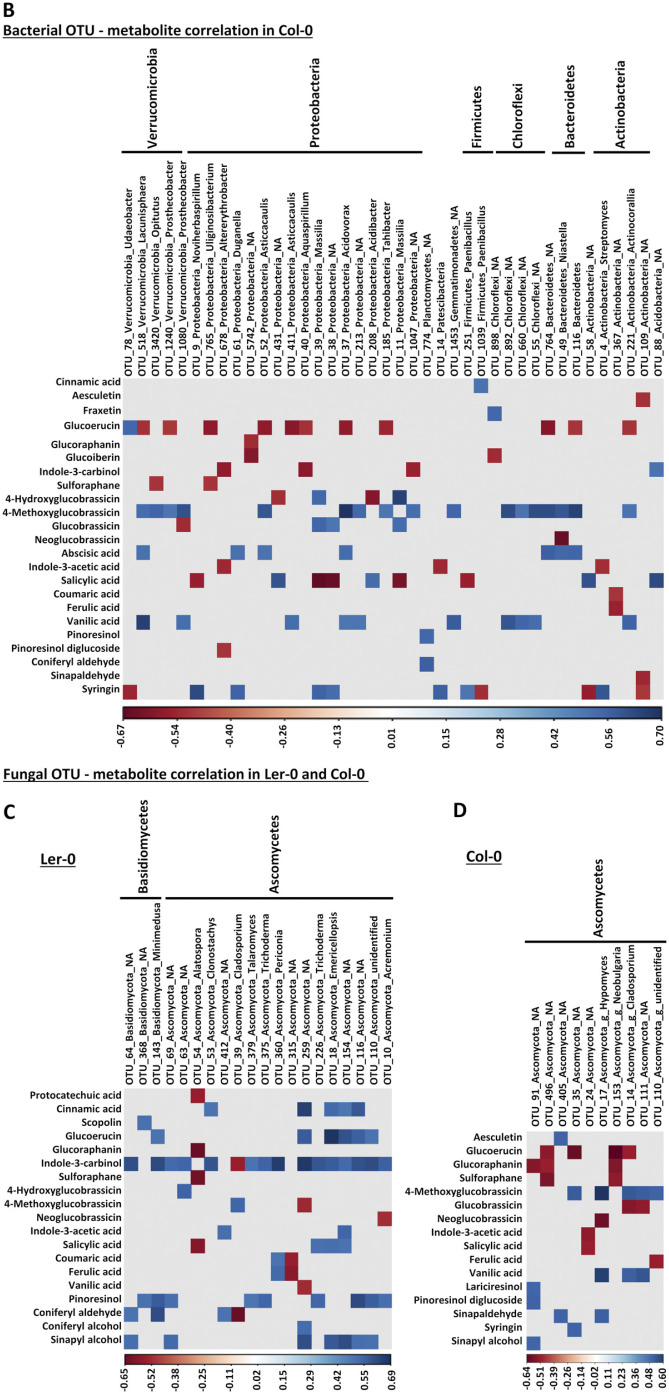
Microbial OTU-metabolite correlations. The bOTU-metabolite correlation in Ler-0 (A) and Col-0 (B). The fOTU-metabolite correlations in Ler-0 (C) and Col-0 (D). All microbiome and metabolite datasets generated for Col-0 and Ler-0 was used in this analysis. Metabolite-OTU associations with strong correlations (−0.4 > r > 0.4 and *P* < 0.01) were visualized (A-C).

Inter-omics analysis was performed using Spearman’s rank correlations of root metabolites and microbial OTU data sets generated from inoculated and noninoculated samples of genotypes, and significant microbial-metabolite correlations were visualized using heatmaps (Fig. 5). The highest numbers of correlations were found in the susceptible Ler-0, both for bOTUs and fOTUs (Fig. 5; Table S10). Strikingly, in Ler-0, several bOTUs belonging to Actinobacteria, and few bOTUs assigned to Chloroflexi, and Firmicutes generally correlated positively with the targeted metabolites. In contrast, bOTUs belonging to Proteobacteria, Verrucobacteria, and a few bOTUs assigned to Acidobacteria, Bacteriodetes, and Planctomycetes negatively correlated with the targeted metabolites (Fig. 5A; Table S10).

**FIG 6:**
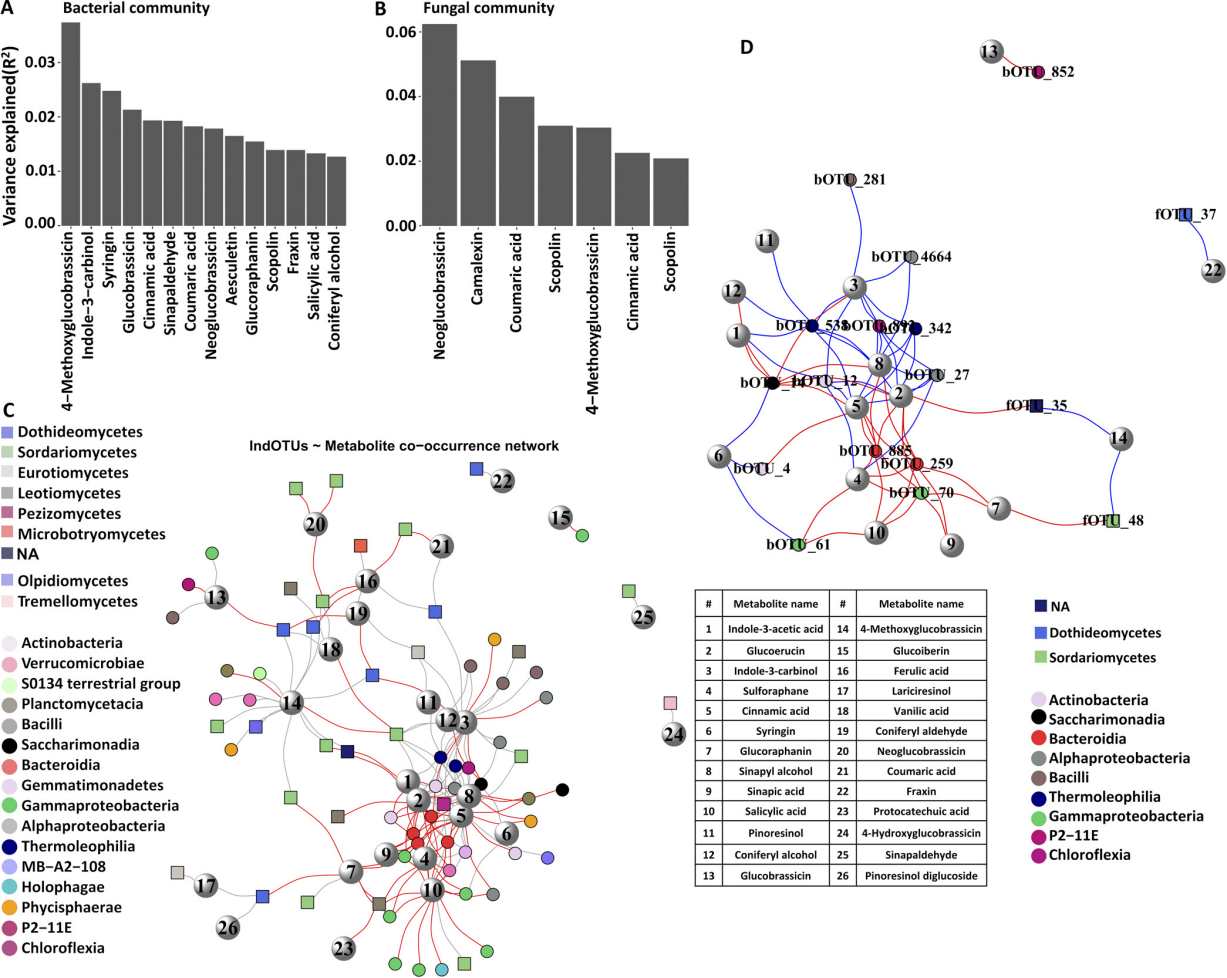
Metabolites with strongest effects and association with important microbial taxa. Metabolites with the highest effects on bacterial (A) and fungal (B) community diversity. Indicator and metabolites co-occurrence network in inoculated and noninoculated Col-0 and Ler-0 (C). Indicator-hubOTU and metabolites co-occurrence network in inoculated and noninoculated Col-0 and Ler-0 (D). Metabolite names corresponding to assigned numbers in C and D are shown in the table.

Cinnamic acid, glucoerucin, indole-3-carbinol, and sinapyl alcohol had the highest numbers of both negative and positive correlations with bOTUs in Ler-0 (Fig. 5A; Table S10). In the Col-0 data set, vanilic acid and 4-methyoxyglucobrassicin were positively correlating with several OTUs, while glucoerucin was negatively correlating with many bOTUs (Fig. 5B; Table S10). A higher relative number of negative metabolite-fOTU correlations were identified in Col-0, and neoglucobrassicin, glucoraphanin, SA, and ferulic acid negatively correlated with fOTUs in both Col-0 and Ler-0 (Fig. 5C, D; Table S10). In the total data set, we found that 4-methoxyglucobrassicin, indole-3 carbinol, syringin, and glucobrassicin had the strongest effects on bacterial communities, each explaining app. 2% to 4% of the variation (Fig. 6A), while neoglucobrassicin, camalexin, and coumaric acid had the strongest effects on fungal communities explaining 4% to 6% of the variation (Fig. 6B).

To test for direct metabolite effects on FOM, we specifically looked at FOM (fOTU1)-metabolite correlations. Correlations were weak but significant for all associations in Ler-0, with neoglucobrassicin and camalexin positively, and scopolin negatively correlating with FOM (Table S11 in the supplemental material). Both scopolin and fraxin displayed minor but significant negative correlations with FOM in Col-0 ().

Next, we examined how indicator OTUs were affected by specific metabolites and found that several of these OTUs were correlating with the metabolites having the strongest general effects on microbial community structures (Fig. 6C; Table S12). For example, 4-methoxyglucobrassicin had mostly positive correlations with indicator OTUs in inoculated Col-0. Similarly, indole-3-carbinol positively correlated with several indicator OTUs, for example, bOTU281 (*Paenibacillus*), bOTU12 (*Nocardioides*), and bOTU27 (Methyloligellaceae), in inoculated Ler-0. Surprisingly, camalexin did not associate with indicator OTUs or hubOTUs, although it had notable overall effects on fungal communities.

Several OTUs that were acting as both indicator and hub OTUs were correlating with specific metabolites (Fig. 6D; Table S12). Strikingly, metabolite and hubOTU correlations were all negative in the inoculated Ler-0 and positive in the noninoculated Ler-0. In inoculated Ler-0, indole-3-acetic acid and sinapic acid negatively correlated with bOTU14 (Saccharimonadales) and bOTU70 (*Leptothrix*), respectively. Indole-3-carbinol correlated negatively with bOTU14 (Saccharimonadales) and positively with bOTU538 (Solirubrobacteraceae), while SA correlated positively with bOTU24 (Gemmatimonadaceae) and negatively with bOTU61 (Duganella). There were also negative correlations between SA and bOTU61, and sinapic acid and bOTU70 (*Leptothrix*) in inoculated Ler-0 (Table S12 in the supplemental material). Cinnamic acid correlated positively with bOTU24 and bOTU27 (Methyloligellaceae) and negatively with several indicator/hub OTUs such as bOTU4 (Streptomyces), bOTU259 (*Niastella*), bOTU885 (Chitinophagaceae), and bOTU70 (Burkholderiaceae). In noninoculated Col-0, bOTU14 (Gemmatimonadaceae) negatively correlated with indole-3-acetic acid and indole-3-carbinol, while 4-Methoxyglucobrassicin positively correlated with fOTU35 (BLAST hit: *Setophoma terrestris*) and fOTU48 (Clavicipitaceae).

## DISCUSSION

The dynamic nature of plant-pathogen interactions require interomics analyses of temporal relationships (11, 36, 37). By following FOM progression over time, we tracked the orchestration of metabolites and root-associated microbiome assembly in two ecotypes of *Arabidopsis* with different FOM susceptibility levels (38). In agreement with previous studies (39–41), we found distinct microbial compositions not only between ecotypes but also among developmental and infection stages.

### FOM invasion differentially affect resistant and susceptible *Arabidopsis* microbiomes.

Microbiome profiling revealed distinct microbial communities in the resistant Col-0 and susceptible Ler-0 after FOM inoculation and also in noninoculated roots. In both ecotypes, the effect of FOM inoculation on microbial communities was strongest at the early time points, which could be attributed to high initial FOM densities resulting from the inoculation. Genotype effects on microbial communities increased with DAI, and although the relative importance of genotype on microbial communities has been shown to decline over time (42), our results showed the opposite trend, especially for the bacterial communities. These genotype response trajectories on the microbiome of inoculated plants could possibly play a role in disease resistance.

The temporal dynamics of indicator OTUs showing high numbers of indicator OTUs at 25 DAI in Col-0, revealed (i) a large diversity of the *Arabidopsis* root-associated microbiota that was distinctively affected by the infection of the host; (ii) a time-dependent assembly of microbial communities as previously reported (39, 40, 43), for example, Edward et al. (39) found that the host-associated microbiota in field grown rice was affected by host developmental stage and plant age; (iii) genotype-specific assembly of microbiota, as also demonstrated by Wagner et al. (44). Notably, the dramatic enrichment of bOTU10 assigned to Pseudomonas protegens in inoculated plants and the strong enrichment of *Streptomyce*s in Col-0 suggest that *Arabidopsis* enriches specific microorganisms to enhance FOM resistance. P. protegens is a plant growth-promoting endophytic bacterium with broad-spectrum antifungal activity (45), while *Streptomyces* is known for its unparalleled synthesis of antibiotics (46) that could contribute to the observed FOM resistance in Col-0 (46, 47). The genus *Flavobacterium* found to be significantly enriched in Col-0 was reported to suppress F. oxysporum in the *Allium fistulosum* microbiome (48). Moreover, the enrichment of numerous taxa in Col-0 at 25 DAI further supports distinct genotype effects on microbiome assembly. Previous studies have also reported a strong enrichment of the bacterial genera Pseudomonas and *Streptomyces* during F. oxysporum infection of chili pepper (*Capsicum annuum* L.) (49), common bean (Phaseolus vulgaris) (50), and also in Fusarium wilt suppressive soils (51, 52). In combination, these results strongly suggest Pseudomonas and *Streptomyces* as highly active in Fusarium induced microbiomes and could potentially be exploited for engineering sustainable Fusarium control strategies.

Microbial communities in Col-0 compared with Ler-0 as demonstrated by stronger network parameters (node degree, hub numbers, and link densities) indicated higher robustness. In contrast, connectance (the proportion of all possible interactions that are realized in a network) was highest in Ler-0, corroborating an earlier study where high connectance promoted Ralstonia solanacearum host colonization (26). In addition, network breakdown (assessed using node degree) during FOM invasion was highest in Ler-0. Altogether, these results support our hypothesis of stronger networks in resistant plants that are contributing to overall pathogen resistance. Also, the positive and negative correlations between bacterial and fungal OTUs support previous studies of cooperative and antagonistic microbial interactions among microbial kingdoms (20, 28). The higher number of negative co-occurrences in inoculated Ler-0 could be due to a rapid colonization by saprophytic bacteria in the root tissues (53) degraded by FOM. We only found few negative co-occurrences between FOM (fOTU1) and bOTUs, and these were observed mostly in noninoculated Ler-0. Most of these bOTUs belonged to Proteobacteria and Actinobacteria, which are known for their prevalence in the *Arabidopsis* microbiome (54), as well as their profound role in disease suppression (52). The differences in co-occurrence network structures underscore the distinct interactions in FOM-inoculated and noninoculated Col-0 and Ler-0 and also explain aspects of host defense (17, 28). The microbial networks further highlighted how indicator species are affected during FOM invasion. Surprisingly, keystone species and indicator species were mostly observed in the networks of the inoculated Col-0, and we speculate that recruitment of these species in the presence of pathogens could serve as an important factor in disease resistance (55).

### The *Arabidopsis* metabolome is distinctively altered in Arabidopsis during FOM attack.

Plant metabolites are strong modulators of microbial communities in general and more specific of pathogen invasion resistance (56). The observed separation of metabolites in the OPLS-DA at individual DAIs and the fact that this coincide with the assembly of host-associated microbiota is a notable indication of omics interdependence (5, 57). The observed differences in iGLS, aGLS, camalexin, and phenylpropanoid concentrations in Col-0 and Ler-0 during infection could contribute to their differential FOM resistances. For example, aGLSs that are having higher inhibitory effects on microorganisms (58) had higher levels and conceivably higher FOM-suppression effects in inoculated Col-0 than in inoculated Ler-0 at 5 DAI. Accordingly, the elevated concentrations of sinapyl aldehyde, syringin, sinapyl alcohol, and coniferyl aldehyde in inoculated plants support previous findings of the suppressive effects of *Arabidopsis* phenylpropanoid derivatives on *Verticillium longisporum* (59, 60).

### Microbial OTUs are affected by specific metabolites in *Arabidopsis*.

There were distinct metabolome-microbiome correlative patterns in Ler-0 and Col-0. Remarkably, we found that most of the analyzed metabolites positively correlated with the bacterial taxa Proteobacteria, Bacteriodetes, Planctomycetes, Acidobacteria, and Verrucomicrobia, and negatively correlated with Actinobacteria, Firmicutes, and Chloroflexi in Ler-0. This finding is compelling and could indicate that host genotypic variation, including factors underlying both disease resistance differentials and chemical diversity affect microbial community assembly (61). We further posit that the unique interomics associations observed in Col-0 and Ler-0 could be explained by the identified missense variants in the resistance genes *Rfo*3 and *Rlp*2 in Ler-0. In Ler-0, both *Rfo*3 and *Rlp*2 have a high number of missense mutations and were also found to be co-regulated with genes involved in metabolic pathways leading to GLS, phenolic metabolites, and phytohormones. We therefore propose that the missense mutations in *Rfo*3 and *Rlp*2 cause altered metabolomes in Ler-0, and that this could likely drive metabolite-microbial OTU associations.

The observation that cinnamic acid and glucoerucin had the highest numbers of positive and negative correlations with indicator/hub OTUs demonstrates their high bioactivity. Importantly, the compounds explaining the highest effects on microbial communities, such as 4-methoxyglucobrassicin, indole-3-carbinol, and syringin showed the strongest correlations with bOTUs, while neoglucobrassicin, camalexin, and coumaric acid were correlating with fOTUs. Both inhibitory and chemoattracting effects of cinnamic acids and GLS has been demonstrated (62–65). The glucosinolates aGLS and iGLS were mostly active against bOTUs and fOTUs, respectively, highlighting differential effects of these metabolites on microbial communities as also observed previously (6, 66). The iGLSs 4-methoxyglucobrassicin and indole-3-carbinol displayed strong positive correlations with some of the bOTUs while the aGLSs glucoerucin, glucoraphanine, and sulforaphane showed strong negative correlations with the indicator taxa Gammaproteobacteria and Bacteroidetes. These results suggests a higher microbial toxicity of aGLSs compared with iGLSs, as reported earlier (67). We also showed that 4-methoxyglucobrassicin correlated positively with the fungal indicator/hubs OTU35 and OTU48 in Col-0. Similarly, Zeng et al. (68), reported 4-methoxyglucobrassicin as a growth stimulator of ectomycorrhizal fungi, while indole-3-carbinol is known for its broad antimicrobial activity against bacteria and yeasts (69). Glucoerucin is also toxic against a number of pathogens (64, 65). Also, sulforaphane which is known for its selective effects toward different microbial taxa (70), negatively correlated with *Niastella* (bOTU259) and *Leptothrix* (bOTU70) and positively correlated with *Nocardioides* (bOTU12) and *Duganella* (bOTU61).

In addition to the GLS effects, we also found differential effects of phenylpropanoids. For instance, sinapyl-alcohol and sinapic acid were negatively correlating with the family Chitinophagaceae in Ler-0. This finding provides evidence of lignin precursors (71, 72) antagonizing a lignin degrading microbial taxa (73, 74), and could thus constitute a modulating mechanism employed to enhance Ler-0 defense. The potential of other phenylpropanoids, for example coumarins, to differentially inhibit both beneficial and pathogenic microorganisms has been reported (57, 75). In addition, the phytohormone SA showed negative correlations with indicator/hub OTUs, suggestive of their modulating effect on the microbiota, thus, corroborating previous studies (10, 76).

Hub microorganisms are important for maintaining network structure and function (28). Interestingly, in our analysis, all identified hub OTUs were also identified as indicator species, reaffirming their importance in the microbiome and for maintaining network stability after pathogen infection. We observed unique metabolite-indicator/hub OTU correlations, mostly positive in noninoculated Ler-0 while being negative in inoculated Ler-0, suggesting that the host uses metabolites to selectively enrich specific microbes under different physiological conditions (77). For instance, the indicator/hubs bOTU259 (*Niastella*) and bOTU538 (Solirubrobacteraceae) had mostly negative and positive correlations, respectively, with metabolites in Ler-0. Solirubrobacteraceae has been found to suppress common scab disease of potatoes (78), while *Niastella* is reported to improve soil health and promote root growth (79, 80). In noninoculated Ler-0, the indicator/hub OTUs *Paenibacillus* (bOTU281), *Nocardioides* (bOTU12), and Methyloligellaceae (bOTU27) exclusively showed positive correlations with metabolites. Interestingly, these taxa are considered ecologically important and are either involved in nitrogen fixation or could be acting as antagonists against pathogens (81, 82). For example, the plant growth-promoting Paenibacillus polymyxa induces host defense responses against F. oxysporum (81, 83). Altogether, these results support our hypothesis of genotype specific metabolome-microbiome interactions primarily due to the differential FOM resistances. Specifically, we speculate that in the lack of a strong direct plant defense response due to the missense variants of resistance genes *Rlp*2 and *Rfo*3, Ler-0 recruits FOM antagonistic microorganisms by synthesizing an array of metabolites to combat progression of the pathogen.

### Conclusions.

This study showed evidence of the dynamic relationships that exist between the plant metabolome and the root-associated microbiome of FOM-inoculated and noninoculated *Arabidopsis* with different FOM susceptibilities. Both the microbiome and the metabolome in the two *Arabidopsis* genotypes were distinct and significantly shifted during FOM infection. Microbial networks in the resistant Col-0 were more robust compared with networks in the susceptible Ler-0 during FOM infection, indicating a role of the microbiome in *Arabidopsis* pathogen resistance. Pseudomonas protegens (bOTU10) and *Rhizobium* (bOTU6) were highly enriched in inoculated samples of both genotypes, suggesting a prominent role of these OTUs in the plant response to FOM infection. The genus *Streptomyces* (bOTU4) was strongly enriched in Col-0 than in Ler-0, suggesting a possible role in resistance against FOM in Col-0. We found distinct associations between metabolites and the bacterial phyla Proteobacteria, Bacteroidetes, Planctomycetes, Acidobacteria, and Verrucomicrobia, and negative correlations with Actinobacteria, Firmicutes, and Chloroflexi in Ler-0, which could be explained by the highly mutated resistance genes *Rfo*3 and *Rlp*2. The GLS 4-methyoxyglucobrassicin, glucoerucin, and indole-3 carbinol, but also phenolic compounds, were correlating with indicator and hub OTUs and were thus highly active in structuring the *A. thaliana* root-associated microbiome. Considering the major role of indicator/hub microbial taxa in overall microbiome composition, we infer that metabolites correlating with these taxa could be pivotal in structuring the *Arabidopsis* root microbiome. It is worth emphasizing that, although correlation-based analyses are not definitively causal, the identified associations deepen our understanding of interomics interactions in resistant and susceptible plant microbiomes. In future studies, it will be interesting to study the possible microbiome-mediated direct effects of *Rfo*3 and *Rlp*2 resistance genes.

## MATERIALS AND METHODS

### Arabidopsis thaliana genotypes and Fusarium isolate.

We used the Arabidopsis ecotypes *Columbia*-0 (Col-0) and *Landsberg erecta*-0 (Ler-0) lines in this study. While Col-0 is a natural accession and maintained as a clean homozygous line, the Ler-0 carries mutations that are caused by X-ray irradiation in the ERECTA gene (31). These accessions have been shown to exhibit distinct root morphologies (84, 85), chemical profiles (86), and disease resistances against F. oxysporum (30). Both accessions were supplied by the Nottingham Arabidopsis stock center (NASC), United Kingdom. FOM isolate 726 (87) was kindly provided by Dr. H. Corby Kistler at USDA ARS CDL-University of MN, USA.

### Experimental design.

Arabidopsis seeds were sown in pots (8 × 8 × 6 cm) containing field soil (fine sand 32.2%, coarse sand 52.8%, humus 4.7%, clay 3%, silt 7.3%) (88) and pH 5.95, collected from the Jyndevad Experimental station (54.9023° N, 9.1511° E), Denmark in 2016. We sowed approximately 20 seeds per each pot. In total, we maintained 100 pots allocated to the two genotypes, (Col-0 and Ler-0), two treatments (FOM-inoculated or water-inoculated, noninoculated), five replicates of each treatment, and destructively sampled at five different time points.

The pots were arranged in trays, loosely covered with plastic wrap, and the seeds were stratified in the dark at 4°C for 3 days. Thereafter, the pots were completely randomized and maintained under greenhouse conditions (16 h light, 8 h dark and 18°C to 23°C) for the entire duration of the experiment. After germination, thinning-out was done, leaving 10 seedlings in each pot. Seedlings were watered (100 mL/pot) 2 times per week and weeds removed regularly upon emergence.

### Pathogen culture and inoculation.

FOM was cultured on sporulation-induced synthetic nutrient-poor agar (SNA) medium: 1 g KH_2_PO_4_, 1 g KNO_3_, 0.5 g MgSO_4_·7H_2_0, 0.5 g KCI, 0.2 g glucose, 0.2 g sucrose, 20 g agar, 1 L distilled water) and incubated 1 week under day/night light conditions at 20°C to 23°C. Mycelial plugs (6 plugs/100 mL from 7-day-old FOM plates) were transferred into a 400 mL sterile liquid carboxymethylcellulose (CMC) medium: 15 g CMC sodium salt (high viscosity, #C5013: Sigma-Aldrich, St. Louis, MO, USA), 1g NH_4_NO_3_, 1g KH_2_PO_4_, 1g yeast extract, 0.5 g MgSO_4_·7H_2_O and 1 L of distilled water and cultured for 3 days at 22°C in the dark with gyratory shaking (125 rpm). We harvested fungal spores by filtering through sterile Miracloth to remove mycelia and centrifuged (4,500 *g*) the filtrate containing FOM spores for 15 min at room temperature to pellet spores. The spores were washed twice with sterile deionized distilled H_2_O, followed by centrifugation at 7,500 *g* for 5 min at room temperature before discarding the supernatant. We resuspended the spore pellet in sterile water and estimated spore concentrations using a hemocytometer before adjustment to 1.1 × 10^6^ spores/mL. The soils surrounding the 2-week-old seedlings were inoculated each with 300 μL of the adjusted spore suspension by carefully pipetting into soils close to the roots of the seedlings (50 pots), while the 50 noninoculated pots received 300 μL distilled water.

### Sample collection.

Root samples were collected at five different sampling times at intervals of 5 days with the first sampling performed at 5 DAI with FOM. Roots were harvested by pressing the sides of each pot to loosen the soil around the roots. Subsequently, each root system was carefully pulled out and shaken gently to remove loosely attached soil, then cut with sterile scissors. Roots with adhering rhizosphere soil of the 10 plants in the individual pots were pooled into sterile 2 mL collecting tubes. The root samples were divided to allow for both metabarcoding and chemical analysis. Samples for chemical analysis were placed into prechilled tubes. Half of the root samples determined for chemical analysis were used in this study, the other half used in another study (38). Shoot (stems and leaves of plants) samples were taken for FOM quantification. Harvested samples were immediately snap-frozen in liquid nitrogen and stored at −20°C (samples for metabarcoding) or −80°C (samples for chemical analysis). Root and shoot samples were lyophilized for 3 days. All lyophilized samples were stored at −20°C prior to downstream processing.

### DNA extraction.

Lyophilized samples were ground using sterile steel beads in a Geno/Grinder2000 (Spex, Metuchen, NJ, USA) at 1,500 rpm for 3 × 30 s. For both root and shoot DNA extraction, we used 250 mg of ground sample. Root DNA extraction was done using the PowerLyzer PowerSoil DNA isolation kit (Mo Bio Laboratories, Carlsbad, CA, USA). Shoot DNA was extracted using the DNeasy Plant minikit (Qiagen, Hilden, Germany) according to the manufacturer’s instructions. The extracted shoot and root DNA samples were stored at − 20°C and subsequently used for FOM quantification or sequencing library preparation.

### FOM quantification in shoots.

We estimated F. oxysporum biomass in inoculated and noninoculated shoot samples, using quantitative PCR (qPCR) using the F. oxysporum specific primers F 5′-CCTGTTCGAGCGTCATTTCA-3′ and R 5′-GAATTAACGCGAGTCCCAACAC-3. The PCR consisted of 2.5 μL template, 0.375 μL each of forward and reverse primers (10 μM stock), 3 μL of water, and 6.25 μL of Bio SyGreen Mix Lo-Rox (PCR Biosystems, Ltd., London, UK). For positive and negative controls, 2 μL of template (dilutions of FOM DNA, or sterile water) were added. Amplification reactions were performed with two replicates per sample using a ViiA 7 real-time PCR system (Life Technologies, CA, USA). Thermal cycling conditions included an initial denaturation at 95°C for 10 min followed by 40 cycles of 95°C for 15 s and 58°C for 1 min. Standards were included in the run using a 10x dilution series of FOM DNA with an initial concentration of 2.05 ng/μL. A standard curve was obtained by plotting the cycle threshold (*C*_T_) values as a function of log_10_ of the amount of fungal DNA added in a 10-fold serial dilution (1 to 10^−9^).

### Library preparation.

For the microbiome analysis, we followed 16S and ITS library preparation procedures as previously described (5). Briefly, the bacterial 16S rRNA V3/V4 amplicon library was generated using the PCR primers (S-DBact-0341-b-S-17/S-d-Bact-0785-a-A-21) (89). For amplification of the fungal internal transcribed spacer 2 (ITS2) region, we used the fITS7 (90) and ITS4 (91) primer pair. Both bacterial and fungal libraries for Illumina MiSeq sequencing were generated by a two-step dual indexing strategy as previously described (5) and sequenced at Eurofins MWG (Ebersberg, Germany). The raw sequence files were deposited at the National Centre for Biotechnology Information (NCBI) sequence read archive with the SRA accession number PRJNA756534.

### Metabolite extraction.

Metabolic pathways of the quantified metabolites are shown (Fig. S1). Prior to metabolite extraction, approximately 5 mg of dried tissue was ground to a fine powder by using a Geno/Grinder 2010. Metabolites from root material were extracted by the addition of 1 mL of 70% (vol/vol) methanol/water solution to the plant material (38). The tubes were vortexed for 20 s before heating at 72°C for 10 min to avoid myrosinase-mediated glucosinolate breakdown (92). Samples were cooled to room temperature and placed in a sonication bath for 5 min. Next, the samples were shaken at 30 rpm for 15 min at 4°C before centrifugation for 5 min at 15,000 g, and transfer of the supernatant into new tubes. The supernatant was diluted in Milli-Q water, filtered through a 0.22-μm KX syringe filter (PTFE 13-mm diameter) (Mikrolab, Aarhus, Denmark) and injected into the LC-MS/MS system.

### Liquid chromatography-tandem mass spectrometry analysis.

Samples were analyzed in multiple reaction mode (MRM) on an Agilent 1260 infinity HPLC system (Santa Clara, CA, USA) connected to an AB Sciex 4500 triple-quadrupole trap mass spectrometer (QTRAP/MS) (AB Sciex, Framingham, USA) equipped with electrospray ionization (ESI) source in negative and positive ion mode. MRM-transitions and compound-dependent parameters are summarized in Table S1. The information with respect to mass spectrometry parameters for multiple reaction monitoring can be found in supplemental material together with additional information about the liquid chromatography-tandem mass spectrometry (LC-MS/MS) method. Chromatographic separation for glucosinolates and plant hormones (negative mode) (Table S1) was performed at 40°C on a reversed-phase Synergi Fusion-RP C18, 80A column (250 mm × 2 mm i.d., 4 μm, Phenomenex) equipped with a Security Guard Cartridge (KJ0-4282, Phenomenex) (93). For compounds related to lignin and lignan biosynthetic pathway (Table S1), plant hormones (positive mode), coumarins, and phenolic acids, the separation was carried out on a Kinetex EVO C18 (150 × 2.1 mm i.d., 5 μm, Phenomenex) protected by a Security Guard ULTRA Cartridge (AJ0-9298, Phenomenex). Further details on the stepwise gradient used in the LC-MS/MS analysis can be found in the supporting information. All data were collected using ABSciex Analyst software (version 1.6.2). Quantitation was performed using ABSciex MultiQuant software (version 3.0.2). Samples were run in randomized order.

### Sequence data and statistical analysis.

Bacterial and fungal sequence reads were analyzed as described earlier (5). Briefly, paired-end reads were demultiplexed for internal barcodes, using Mr_Demuxy using command pe_demuxer.py (https://pypi.org/project/Mr_Demuxy/). Subsequently, paired-end reads were assembled and joined reads were processed, using vsearch v.2.6 (94). Primers were removed, using cutapdapt (95). Dereplication, chimera screening, and clustering of sequences were performed using vsearch v.2.6 (94). Extraction of fungal ITS reads was carried out prior to clustering, using ITSx extractor version 1.0.6 (96). Taxonomy assignments were performed using the SILVA 132 (97) and UNITE (v7.2) (98) reference databases, respectively, for bacteria and fungi, in QIIME (v1.9) using assign_taxonomy.py (99). Unassigned OTUs at kingdom level or OTUs assigned as chloroplast or mitochondrial sequences were removed. Also, OTUs found in less than three samples in the total data set were removed.

Statistical analyses and data visualizations were carried out in R v4.0.5 (100), using vegan (v2.5.7) (101), phyloseq (v1.34.0.) (102), ggplot2 (v3.3.2) (103) packages. Before diversity analysis, samples with less than 1,000 reads were removed from the data sets. OTU tables were rarified 100 times at a depth of 1,000 reads for both data sets, and the mean of the diversity estimates of 100 trials was used to estimate each alpha diversity metric (observed and Shannon diversity). Significant differences between alpha diversities were evaluated, using Kruskal-Wallis rank sum test. OTU tables were transformed to relative abundances (RAs) prior to beta diversity analysis. Bray-Curtis dissimilarity matrices were visualized, using unconstrained principal coordinates analysis (PCoA), and permutation analysis of variance (PERMANOVA) was performed for both bacterial and fungal communities, using the “adonis” function from the “vegan” package. We performed indicator species analysis, using the *labdsv* function in R (104) to determine abundance differentials of the bacterial (bOTUs) and fungal OTUs (fOTUs) associated with inoculated and noninoculated samples of Col-0 and Ler-0. Data sets were partitioned and analyzed separately for Col-0 and Ler-0 for identification of indicator OTUs. Highly significant OTUs (*P* ≤ 0.01) with indicator values > 0.4 were considered indicator species (105).

We performed differential abundance analysis of microbial OTUs based on negative binomial distribution between inoculated and noninoculated data sets Col-0 and Ler-0 using “DESeq2” R package (102, 106).

### Microbial network analysis.

Microbial interaction patterns in roots of noninoculated and inoculated samples of Col-0 and Ler-0 were examined by Spearman’s rank correlation analysis. We pooled bacterial and fungal data sets generated for the 5 time points in the construction of the respective networks. Next, bacterial and fungal OTUs were pooled and normalized, using trimmed mean of M values (TMM) method using the BioConductor package EdgeR in R (107). For microbial network construction, we used OTUs that were present in at least 10 samples with Spearman’s rank correlations > 0.7 for positive correlations and < −0.7 for negative correlations, and *P* values < 0.001. The correlated OTUs were visualized in networks with OTUs set as nodes and correlations as edges. OTUs that were identified in the indicator analysis and appeared in the co-occurrence analysis were shown as larger nodes. Network properties were computed, using the “igraph” package with defined parameters as described in (5). We used Spearman’s rank correlations to determine associations between fOTU1 and bOTUs.

### Statistical analysis for targeted metabolomics.

The absolute concentrations (μg/g) from the targeted metabolomics analysis were subjected to the time‐series and two‐factor data analysis by MetaboAnalyst 4.0 (108) with Bonferroni correction to determine the level of significance between the factors and their interaction. Bonferroni-corrected and adjusted *P* values less than 0.05 were considered significant. SIMCA-P (ver. 15.0.2, Umetrics AB, Umeå, Sweden) was used to perform orthogonal partial least square-discriminant analysis (OPLS-DA) to determine the metabolites that were contributing to the variation between treatment groups at different infection stages. OPLS-DA models were performed on cubic root-transformed and Pareto-scaled data to identify clustering behavior related to treatment groups. OPLS-DA models were validated by correlation (R2) and predictability (Q2) parameters. In addition, a permutation test (*n* = 100) was performed to validate the robustness and overfitting of the OPLS-DA models. The assessment of the validated models was performed by inspecting the intercept of the permutation plot (permQ2). The significance of the OPLSDA model was assessed by the cross-validated analysis of variance (CV-ANOVA). A heatmap was generated with MultiExperiment Viewer application (109) and visualized, showing log_2_-transformed significant differences between FOM inoculated plants and control. Fold changes between inoculated and noninoculated plants were calculated and Student’s t-tests for two group-comparisons were performed by SigmaPlot software (version 11.0). The nonparametric Mann-Whitney Rank Sum test was used when the data violated the assumption of normality. Metabolites with a threshold of fold change (FC) > 1 as well as *P* < 0.05 were considered significant. Bar plots were generated, using ggplot2 in R to observe variation in metabolite concentrations shown as means ± standard error.

### OTU-metabolite correlation analysis.

We examined metabolite microbial OTU associations by performing Spearman’s correlation analysis, using the rcorr function. Prior to the analysis, we filtered low abundance OTUs by removing OTUs occurring in less than four samples and less than 50 read counts followed by a transformation to relative abundance. The metabolome data were also transformed to fit the normal distribution pattern. Metabolite-OTU associations with strong correlations (*ρ* > 0.4 or < −0.4 and *P* < 0.01) were visualized in heatmaps. In addition, relative abundance patterns of the correlated metabolite OTUs across different DAI were visualized, using bubble plots.

### Resistance gene-metabolome relationship.

To assess the relationship between FOM resistance genes and metabolites, we analyzed genes involved in Fusarium resistance using transcriptome data downloaded from The Botany Array Resource (110) and ATTED-II (111). Genes with a Pearson’s correlation coefficient larger than 0.75 and a mutual Rank (111) of at least 2.2 were defined as co-expressed genes. Metascape (112) was used for pathway enrichment analysis. The *Arabidopsis* data from the 1001 Genomes Consortium (113) were used for gene polymorphism analysis of the ecotypes.

### Data availability

The MiSeq paired end reads for bacterial 16s rRNA gene (V3 to V4) and fungal ITS2 regions have been deposited in NCBI SRA database using accession code PRJNA756534. Sequence processing in QIIME and data analysis in R were performed using the pipeline and scripts from Kudjordjie et al. (5).
